# Association of fully branded, standardized packaging and limited flavor and brand descriptors of e‐liquids with interest in trying products among youths in Great Britain

**DOI:** 10.1111/add.16763

**Published:** 2025-01-13

**Authors:** Eve Taylor, Erikas Simonavičius, Matilda Nottage, Ann McNeill, Deborah Arnott, Hazel Cheeseman, David Hammond, Jessica Reid, Pete Driezen, Kimberly D'Mello, Katherine East

**Affiliations:** ^1^ Department of Behavioural Science and Health University College London London UK; ^2^ Department of Addictions Institute of Psychiatry, Psychology and Neuroscience, King's College London London UK; ^3^ Action on Smoking and Health (ASH) London UK; ^4^ School of Public Health Sciences University of Waterloo Waterloo Canada; ^5^ Department of Primary Care and Public Health Brighton & Sussex Medical School Brighton UK

**Keywords:** e‐liquid, nicotine, packaging, policy, vaping, youth

## Abstract

**Background and aims:**

Many vaping products feature bright colors and novel brand names and flavor descriptors, which may appeal to youth. We measured the strength of the associations between e‐liquid packaging design (branded, white standardized or white standardized limiting brand and flavor descriptors) and perceived peer interest in trying the e‐liquids among youth.

**Design:**

A between‐subjects online experiment.

**Setting:**

The Action on Smoking and Health Smokefree Great Britain (GB) Youth 2021 online survey.

**Participants:**

Participants included 1628 youth aged 11–18, 51.9% female, 71.8% socioeconomic status ABC1 (the three highest Market Research Society grades).

**Measurements:**

Participants were randomized to view a set of three images of e‐liquids from one of three packaging conditions: (1) fully branded (control), (2) white standardized with usual brand names and flavor descriptors or (3) white standardized with coded brand names and limited flavor descriptors. Participants were asked which e‐liquid they thought people their age would be most interested in trying and could select a product, ‘none of these’, or ‘do not know’. Multinomial logistic regression models were used to test associations between selecting ‘none of these’ (‘no interest’) versus any product (‘interest’) or ‘do not know’ and packaging condition. Analyses were adjusted for sex, age, socioeconomic status, vaping status and smoking status.

**Findings:**

Compared with fully branded packaging (22.7%; reference category), youth had higher odds of reporting no perceived peer interest in trying e‐liquids in standardized packs with brand codes and limited flavor descriptors [30.3%, adjusted odds ratio (AOR) = 2.07, 95% confidence interval (CI) = 1.53–2.79], but not standardized packs with usual descriptors (23.1%, AOR = 1.21, 95% CI = 0.89–1.65). Youth had higher odds of reporting no perceived peer interest in e‐liquids in white standardized packs with brand codes and limited flavor descriptors (30.3%, AOR = 1.87, 95% CI = 1.29–2.16, *P* < 0.001) compared with standardized packs with usual descriptors (23.1%; reference category).

**Conclusion:**

Standardized e‐liquid packaging that limits flavor and brand descriptors may reduce the youth appeal of e‐liquids.

## INTRODUCTION

In Great Britain (GB), use of e‐cigarettes (vaping) at least once a month has increased among youth age 11 to 17, from 3.3% in 2021 to 7.2% in 2024 [1]. E‐cigarette and e‐liquid branding often contain elements that appeal to youth, such as cartoons [2, 3, 4, 5]. Youth also report finding the range of available e‐cigarette flavors attractive [6] and have higher appraisal and receptivity scores for flavored e‐liquid packaging compared to unflavored [7]. E‐liquid brands often use images, sensory descriptors and conceptual names (e.g. ‘blue voltage’ or ‘solar’) to describe flavors on their packaging [8] and on‐line advertising [9, 10]. Conceptual names are popular among youth [11], although youth have been found to prefer names with characterizing flavors [12].

In GB, e‐cigarettes and e‐liquids that contain nicotine are regulated by the Tobacco and Related Products Regulations [13]. For example, vapes and e‐liquids cannot be sold to youth under the age of 18, e‐liquid bottles should not exceed a maximum volume of 10 mL, nicotine strength is limited to a maximum concentration of 20 mg/mL and packaging must display nicotine content, ingredients and a nicotine health warning label [7], and all broadcast media of nicotine vaping products is banned. Other forms of marketing are regulated in GB for e‐cigarette products through the Advertising Standards Authority [14]. There are also regulations on the claims and themes that can be presented on packaging. For example, packaging cannot include claims that these products provide any health or lifestyle benefits or have vitalizing, energizing, healing, rejuvenating, natural or organic properties [13]. These regulations, however, are difficult to define and enforce and do not currently restrict the use of colors and cartoons. In November 2024, the United Kingdom (UK) government announced plans to regulate the packaging and flavors of e‐cigarettes and e‐liquids [15]. However, details of the regulations have not yet been announced. Therefore, packaging is an important source of point‐of‐sale and peer‐to‐peer advertising for e‐cigarette and e‐liquid brands.

Previous research indicates that standardized cigarette packaging of tobacco cigarettes in olive green packs can reduce their appeal to youth [16], and similar findings have recently been observed for e‐cigarettes [17]. Using the Action on Smoking and Health (ASH) 2021 GB Adult and Youth surveys, our prior work found that standardized e‐cigarette packaging can reduce appeal to youth without reducing appeal to adults [17], such that fewer youth from GB reporting interest in trying e‐cigarette products in standardized green (the same color as tobacco cigarette packaging in GB) or white packs compared to fully branded packs. Similar effects of standardized packaging for e‐liquids have been found among youth in England, Canada and the United States (US) [18].

Before the introduction of standardized packaging for cigarettes, flavor descriptors, such as ‘mild’ or ‘smooth’, were banned [13]. Brand identity has been shown to be important to people who smoke [16], with youth reporting that brand names can be appealing and encourage purchase [17]. Youth are also reported to perceive certain cigarette brand names as cool and sophisticated even when in standardized packs [19]. Cigarette packs with brand descriptors removed are also perceived by youth as less appealing, less cool and less glamorous than packs with descriptions such as ‘slim’ and ‘pink’ [20]. Brand names have also been found to influence cigarette taste and risk perceptions [16]. Similarly, for vapes, youth have reported more favorable attitudes when devices were referred to by brand names rather than as generic e‐cigarettes [21]. Reducing brand and flavor descriptions on e‐liquids could also reduce brand identity, and in turn appeal, to youth, but there is little research in this area. Therefore, our research question was: what is the strength of the associations between e‐liquid packaging design (branded, white standardized or white standardized limiting brand and flavor descriptors) and perceived peer interest in trying the e‐liquids among youth in GB?

## METHODS

### Data source

Data were from the on‐line 2021 ASH Smokefree GB Youth Survey (ASH‐Y) of tobacco and vaping product use among youth age 11 to 18 years in GB. This survey is conducted annually by ASH and is drawn from an existing on‐line panel maintained by YouGov. Active sampling was used, whereby restrictions were put in place to ensure only those who were selected from a YouGov panel of registered users were allowed to take part [13]. Respondents were invited by email to participate in the on‐line survey, which took place between 25 March 2021 and 16 April 2021. Informed consent was provided either by the parents of those 11 to 15 years or by those individuals 16 to 18 years. Ethical approval for the analyses in this article was not required because this study involved secondary analysis of pre‐existing data, in line with King's College London's policy.

### Design

**FIGURE 1:**
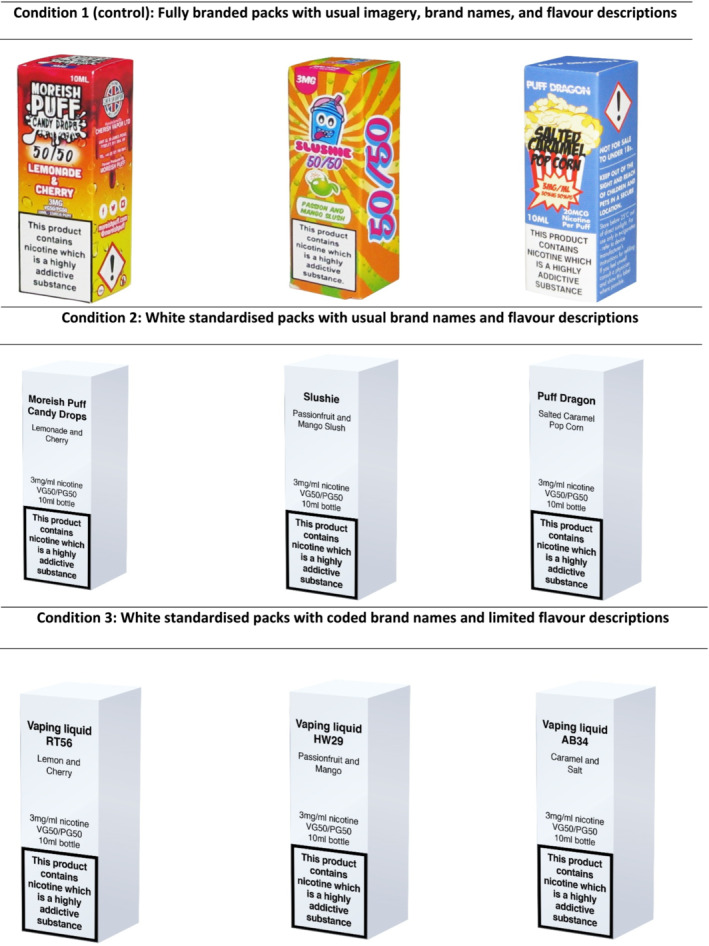
E‐liquid packs by experimental condition.

A ‘between‐subject’ experiment was included on completion of the ASH‐Y to compare perceptions of e‐liquid pack images that were (1) fully branded (i.e. usual colors, images, brands and flavor descriptors; control); (2) digitally altered to be in white standardized packs with usual descriptors (i.e. no colors or imagery, but usual brands and flavor descriptors); or (3) white standardized with coded brand names and limited flavor descriptions (Figure 1). Packaging was standardized in white, as opposed to the green/brown Pantone 448C used for standardized tobacco cigarette packaging in the United Kingdom, to ensure a distinction between e‐liquids and tobacco cigarettes, consistent with prior work in this area [17, 18, 22]. The experimental design was based on previous work evaluating the effects of tobacco cigarette packaging [20, 23] and e‐cigarette packaging [18, 22].

Participants were randomized, using simple randomization, to one of three experimental conditions, in which they viewed a set of three images of e‐liquids in packaging that was either: (1) fully branded; (2) white standardized with usual descriptors (i.e. no colors or imagery, but usual brands and flavor descriptors); or (3) white standardized with coded brand names and limited flavor descriptions (Figure 1). Within each condition, participants viewed three different brands of e‐liquids with three different flavors; brands and flavors were constant across conditions. As there was no publicly available data on the popularity of e‐liquids brands in the United Kingdom, brands were chosen to be representative of a range of flavors and designs available on the UK market. At the time of this study, tank devices that need to be refilled with e‐liquids were the most popular device type among youth [24].

### Participants

The initial ASH‐Y survey was completed by *n* = 2513 youth. Youth were then re‐contacted and asked if they would like to participate in this experiment. Of the initial ASH‐Y sample (*n* = 2513), 66% agreed to take part in the e‐liquid experiment (*n* = 1654). Respondents who reported ‘prefer not to say’ for the outcome (*n* = 12) or ‘don't want to say’ for any covariates (*n* = 14) were excluded from the sample, resulting in a final analytic sample of *n* = 1628 respondents.

## MEASURES

The questionnaires were developed by ASH to monitor smoking and vaping behaviors among youth [1]. Table S1 shows the measures used and their coding.

### No perceived peer interest in trying e‐liquid products (outcome)

Respondents were shown a set of three images of e‐liquid packs based on experimental condition (Figure 1) and asked, ‘Which of these products do you think people your age would be most interested in trying?’. Participants could select one of the three brands, ‘none of these’, ‘do not know’ or ‘prefer not to say’. Response options were coded as ‘interest in trying’ if any of the three brands were selected, ‘no interest in trying’ if ‘none of these’ was selected or ‘do not know’ (previous analysis of these data indicated a significant proportion of ‘do not know’ responses to questions about standardized packaging [17]) (Table S1). Respondents who selected ‘prefer not to say’ were excluded (*n* = 12). Participants were asked about perceived peer interest, rather than their own interest, because of the ethical implications of asking youth who may not be familiar with vaping about interest in trying vaping products.

### Vaping status

Respondents were asked, ‘Have you ever heard of e‐cigarettes? They are also sometimes called vapes, shisha pens or electronic cigarettes’. Those who responded ‘yes’ were asked ‘Which ONE of the following is closest to describing your experience of e‐cigarettes?’ with available responses ranging from ‘I have never used an e‐cigarette’ to ‘I use e‐cigarettes every day’. Response options were coded into three categories: ‘never used’ (youth who were not aware of vapes or had never vaped), ‘tried/former vaping’ (youth who had only tried vaping or youth who used to vape but do not currently vape) and ‘currently use’ (youth who vaped at least monthly). Respondents who reported ‘don't want to say’ were excluded (Table S1).

For sensitivity analyses, responses were coded ‘ever used’ (tired/former use and current use), and ‘never used’.

### Smoking status

Respondents were asked to report which statement best applied to their experience with cigarettes, ranging from ‘I have never smoked cigarettes, not even a puff or two’ to ‘I usually smoke more than six cigarettes a week’. Response options were coded into three categories: ‘never smoked’ (youth who had never smoked), ‘tried/former smoking’ (youth who had only tried smoking or used to smoke but do not currently smoke) and ‘currently smoke’ (youth who reported currently smoking). Respondents who reported ‘don't want to say’ were excluded (Table S1).

For sensitivity analyses, responses were coded ‘ever used’ (tried/former use and current use), and ‘never used’.

### Socio‐demographic covariates

Covariates were sex (male, female), age group (11–15, 16–18 years) and socio‐economic background (ABC1, C2DE; Table S2) [17]. Social grade was based on the occupation of the chief income earner in the household and was asked of the parents of those participants age 11 to 15, and directly of those participants age 16 to 18.

## ANALYSES

Analyses were not pre‐registered and results should be considered exploratory.

A multinomial logistic regression model was fit to compare reporting ‘interest in trying’ (reference group), ‘no interest in trying’, and ‘do not know’ between the three packaging conditions (branded (reference group), white standardized, white standardized limiting brand and flavor descriptors). Analyses were repeated changing the outcome reference group to ‘no interest in trying’ and also the predictor reference group to ‘white standardised packaging’. Interactions between packaging condition and vaping status, and packaging condition and smoking status, were then added to multinomial models. All analyses were adjusted for sex, age group, social grade, vaping and smoking status. Unweighted data were used because the conditions were randomized.

Because of small cell counts for current smoking and current vaping, sensitivity analyses were conducted where smoking and vaping status were collapsed into two categories (ever use and never use) for interactions between packaging condition and vaping or smoking status.

## RESULTS

The sample was comprised of 48.1% males and 51.9% females. Over half of the sample was age 11 to 15 years (56.5%) and 43.5% age 16 to 18 years. The majority were from a higher socio‐economic background (ABC1; 71.4%) (Table 1).

**TABLE 1:** Participant characteristics ASH‐Y 2021, overall and by experimental condition (*n* = 1628).

	Total	Branded packaging	White standardized with brand codes and limited flavor descriptors	White standardized packaging with usual descriptors
	% (*n*)	% (*n*)	% (*n*)	% (*n*)
Total	100 (1628)	33.0 (538)	33.7 (548)	33.3 (542)
Sex				
Male	48.1 (774)	48.1 (259)	46.9 (257)	47.6 (258)
Female	51.9 (854)	51.9 (279)	53.1 (291)	52.4 (284)
Socio‐economic status				
C2DE	28.6 (466)	29.4 (158)	27.0 (148)	29.5 (160)
ABC1	71.4 (1162)	70.6 (380)	73.0 (400)	70.5 (382)
Age, years				
11–15	56.5 (922)	57.1 (307)	56.0 (307)	56.8 (308)
16–18	43.5 (706)	42.9 (231)	44.0 (241)	43.2 (234)
Vaping status				
Never^a^	86.2 (1403)	86.8 (467)	85.6 (469)	86.1 (467)
Tried/former	9.7 (158)	9.9 (53)	10.4 (57)	8.9 (48)
Current	4.1 (67)	3.3 (18)	4.0 (22)	5.0 (27)
Smoking status				
Never	83.9 (1366)	87.4 (470)	82.9 (454)	81.5 (442)
Tried/former	11.7 (191)	9.6 (52)	12.2 (67)	13.3 (72)
Current	4.4 (71)	3.0 (16)	4.9 (27)	5.2 (28)

*Note*: All data are unweighted.

Abbreviation: ASH‐Y, Action on Smoking and Health Smokefree GB Youth Survey 2021.

^a^ Includes respondents who had never heard of e‐cigarettes.

Just under half (48.2%) selected one of the e‐liquids shown when asked which product people their age would be interested in trying, just over one‐quarter (25.4%) selected ‘none of these’ (hereafter referred to as ‘no interest’) and 26.4% selected ‘do not know’ (Table 2).

**TABLE 2:** Multinomial logistic regression model of the associations between reporting no perceived peer interest in trying or do not know and e‐liquid packaging condition, ASH‐Y 2021 (*n* = 1628).

	Interest in trying any e‐liquid displayed (ref)	No interest in trying any e‐liquid displayed			Do not know		
	% (*n*)	% (*n*)	AOR (95%CI)	*P*	% (*n*)	AOR (95%CI)	*P*
Total	48.2 (784)	25.4 (414)			26.4 (430)		
Packaging condition							
Branded packaging (control)	56.3 (303)	22.7 (122)	1	ref	21.0 (113)	1	ref
White standardized packaging with usual descriptors	48.9 (265)	23.1 (125)	1.21 (0.89–1.65)	0.214	28.0 (152)	1.62 (1.20–2.19)	0.002
White standardized with limited flavor and coded brand descriptors	39.2 (215)	30.3 (166)	2.07 (1.53–2.79)	<0.001	30.5 (167)	2.27 (1.67–3.07)	<0.001
Sex							
Male	41.9 (324)	28.6 (221)	1	ref	29.6 (229)	1	ref
Female	53.7 (459)	22.5 (192)	0.62 (0.48–0.79)	<0.001	23.8 (203)	0.62 (0.49–0.79)	<0.001
Socio‐economic status							
C2DE	42.9 (200)	26.6 (124)	1	ref	30.5 (142)	1	ref
ABC1	50.2 (583)	24.9 (289)	0.85 (0.64–1.11)	0.227	25.0 (290)	0.73 (0.56–0.95)	0.019
Age, years							
11–15	41.8 (385)	29.4 (271)	1	ref	28.9 (266)	1	ref
16–18	56.4 (398)	20.1 (142)	1.67 (1.29–2.16)	<0.001	23.5 (166)	1.41 (1.09–1.81)	0.008
Vaping status							
Never^a^	45.0 (632)	27.4 (384)	1	ref	27.6 (387)	1	ref
Tried/former	65.8 (104)	13.3 (21)	0.42 (0.24–0.72)	0.002	20.9 (33)	0.76 (0.47–1.22)	0.262
Current	70.1 (47)	11.9 (8)	0.29 (0.12–0.72)	0.008	17.9 (12)	0.76 (0.35–1.65)	0.488
Smoking status							
Never	45.2 (617)	26.7 (365)	1	ref	28.1 (384)	1	ref
Tried/former	62.8 (120)	17.3 (33)	0.74 (0.47–1.17)	0.196	19.9 (38)	0.60 (0.38–0.93)	0.022
Current	64.8 (46)	21.1 (15)	1.30 (0.61–2.75)	0.495	14.1 (10)	0.45 (0.20–1.02)	0.056

*Note*: Analyses were adjusted for sex, age, socio‐economic status, vaping status and smoking status. See Supporting information for details on the coding of socio‐economic status. All data are unweighted.

Abbreviations: AOR, adjusted odds ratio; ASH‐Y, Action on Smoking and Health Smokefree GB Youth Survey 2021.

^a^ Includes respondents who had never heard of e‐cigarettes.

**FIGURE 2:**
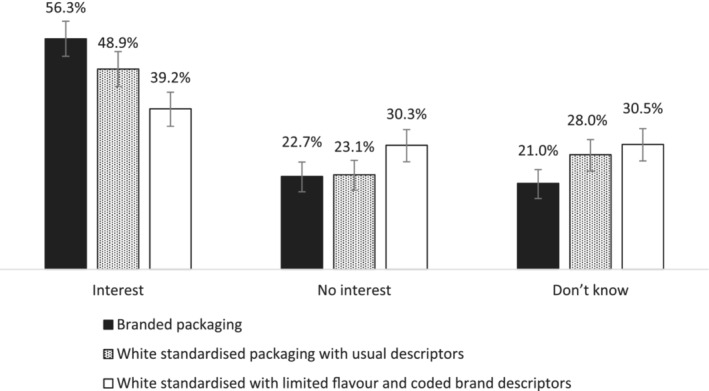
Responses to e‐liquids pack images among youth aged 11 to 18, by experimental condition; ASH‐Y 2021.

Youth who were shown white standardized packaging with brand codes and limited flavour descriptions (30.3%), but not white standardized packaging with usual descriptions (23.1%), had significantly greater odds of reporting that peers would not be interested in trying (‘interest in trying’ as reference) any of the e‐liquids shown, compared to those in the fully branded packaging condition (22.7%) (Table 2, Figure 2). Comparing the two white standardized packaging conditions, youth were significantly more likely to report no peer interest in trying standardized packs with brand codes and limited flavor descriptors [30.3%, adjusted OR (AOR) = 1.87, 95% CI = 1.29–2.16, *P* < 0.001] compared to standardized packaging with usual descriptors (23.1%) (Figure 2).

Youth who were shown white standardized packaging with usual descriptors (28.0%), and with brand codes and limited flavor descriptors (30.5%), were significantly more likely to report do not know (‘interest in trying’ as reference) compared to those in the fully branded packaging (21.0%) (Table 2, Figure 2). Comparing the two white standardized packaging conditions, reporting ‘do not know’ was significantly higher among youth shown white packs with brand codes and limited flavor descriptors (30.5%, AOR = 1.40, 95% CI = 1.05–1.81, *P* = 0.024) compared to those shown white packaging with usual descriptors (28.0%) (Figure 2).

When adjusting the reference category to ‘no interest’, youth were significantly less likely to report that peers would be interested in trying packs in white standardized packaging with brand codes and limited flavour descriptors (39.2%, AOR = 0.48, 95% CI = 0.36–0.65, *P* < 0.001), compared to those shown fully branded packaging (56.3%). Youth were also significantly less likely to report that peers would be interest in trying packs in standardized white packs with brand codes and limited flavor descriptors (39.2%, AOR = 0.59, 95% CI = 0.44–0.79, *P* < 0.001) compared to standardized white packs with usual descriptors (48.9%). There was no significant difference in interest between fully branded packs (56.3%) and standardized packs with usual descriptors (48.9%, AOR = 0.82, 95% CI = 0.61–1.12, *P* = 0.214).

When examining interactions, there were no significant interactions between packaging condition and vaping status (χ^2^ = 14.86, *P* = 0.062), or smoking status (χ^2^ = 3.93, *P* = 0.863) (Figures S1–S2). When vaping and smoking status were collapsed for sensitivity analyses into ever and never use, interactions were significant for vaping (χ^2^ = 16.13, *P* = 0.041) and smoking (χ^2^ = 10.11, *P* = 0.039). Youth who had never smoked had significantly greater odds of reporting that peers would not be interested in trying e‐liquids in standardized packs with limited flavor and coded brand descriptors (33.3%), but not standardized packaging with usual descriptors (24.7%), compared to branded packs (22.3%). Cell counts for youth who had ever smoked were too small to accurately interpret (Table S3). Youth who had never vaped had significantly greater odds of reporting that peers would not be interested in trying e‐liquids in standardized packs with limited flavor and coded brand descriptors (33.9%), but not standardized packs with usual descriptors (25.1%), compared to branded packs (23.1%). Cell counts for youth who had ever vaped were too small to accurately interpret (Table S4).

## DISCUSSION

Overall, youth, of whom the majority were under the age of legal sale of vaping products in the United Kingdom, reported that people their age would not be interested in trying e‐liquids displayed in white standardized packaging with limited flavor descriptions and coded brand descriptions, compared with fully branded packaging. ‘Do not know’ responses were greater among youth who viewed standardized white packaging and standardized white packaging with limited flavor and coded brand descriptions compared to branded packs.

Our findings on standardized packaging are broadly consistent with previous findings on packaging of e‐cigarette starter kits among 11‐ to 18‐year‐olds [17] and e‐liquid packs among 16‐ to 19‐year‐olds [18], and with previous literature on tobacco packaging [16]. The findings concerning the combination of limiting flavor descriptions and using coded brand names are also consistent with findings from tobacco cigarettes with coded brand names [25] and flavor descriptors [16], and suggest that these restrictions are important when standardizing packaging.

Interactions reported that among youth who had never smoked and or vaped, there was reduced peer interest in limited flavor descriptions and coded brand descriptions, compared with fully branded packaging. This is notable, because it is important to dissuade youth who have never vaped and or smoked from trying e‐cigarette products. Sample sizes, however, were too small to accurately interpret the effect of standardized packaging among youth you had ever smoked and or vaped.

Just under half of youth reported perceived peer interest in trying any of the e‐liquids displayed, which is broadly consistent with positive social norms toward vaping among youth in England [26]. In November 2024, the UK government announced that it will introduce powers to make regulations to restrict vape packaging, product presentation and restrict flavors [15]. Our findings suggest that restricting flavor and brand descriptors alongside standardizing packaging on nicotine e‐liquids reduces the appeal of products to youth, indicating that this could be a possible policy direction to reduce vaping products' appeal to youth. ‘Do not know’ responses were also seen to increase for both standardized packaging conditions, suggesting that removing some branding elements and descriptors makes youth less able to distinguish between products.

It is unclear how regulating brand and flavor descriptors of vaping products would affect perceptions of vaping, smoking and subsequent product use among adults and youth. Misperceptions of e‐cigarette harm relative to tobacco cigarettes are common among youth and adults, especially among those who smoke [27, 28] and can deter people from switching from smoking to vaping [24]. Previous research has found that e‐liquids that were presented in standardized white or olive packaging were more likely to be perceived as equally or more harmful than smoking compared to fully branded packs among youth [18]. Moreover, it also found that e‐liquids in standardized white and standardized olive packs were less likely to be perceived as not at all harmful than e‐liquids in branded packs [18]. Therefore, care must be taken for future packaging regulation to not inflate inaccurate harm perceptions, and, in turn, dissuade people who smoke from using e‐cigarettes to help them quit smoking. However, our previous research suggests that removing branded elements from packaging would most likely not impact adult smokers' interest in trying vaping products [17].

There are some limitations to this research. The survey measure asked respondents about perceived interest in trying the vapes displayed among people their age; therefore, responses did not represent participants' own interest, which may be different. Participants were asked which product peers would be most interested in. This wording may have unintentionally introduced bias by inferring that peers would be interested in one of the products, therefore, increasing the likelihood of youth selecting a product rather than reporting no interest. Additionally, the sample was skewed toward youth from a higher socio‐economic status (SES). Although SES was not associated with overall appeal in this study or previous research on standardized cigarette packaging [29], SES can influence warning label effectiveness [30]. Therefore, future research needs to investigate if standardized packaging of vaping products would produce similar effects across different SES backgrounds. Moreover, a third of the youth invited to participate declined, which may have introduced bias into the sample. There were some slight variations in packaging elements across conditions. For example, the branded Slushie ‘Passion and mango slush’ was changed to ‘Passionfruit and mango’ in the white standardized pack with the usual brand name and flavor description condition. Notably, the branded Puff Dragon bottle showed 10 mg/mL of nicotine, which was changed to 3 mg/mL in the standardized versions. Although it is important to consider the possible impact of variations in nicotine concentration labelling, previous research found that youth have little understanding of the strength of e‐liquids [31], and nicotine strength does not affect the appeal of e‐liquids [18], suggesting that this nicotine concentration change likely had little effect on perceived peer interest. Finally, this research was conducted in 2021, before the rapid rise in youth vaping or the introduction of novel disposable products.

## CONCLUSIONS

Standardized e‐liquid packaging, which also limits flavor and brand descriptors, may reduce the appeal of e‐liquids to youth compared to fully branded packs that are currently on the market. These findings highlight the significance of flavor and brand descriptions on vaping product packaging and emphasize the need for additional research to explore their impact on adults who smoke.

## AUTHOR CONTRIBUTIONS

**Eve Taylor:** Conceptualization (lead); formal analysis (lead); funding acquisition (equal); investigation (lead); methodology (lead); writing—original draft (lead). **Erikas Simonavičius:** Methodology (supporting). **Matilda Nottage:** Methodology (supporting). **Ann McNeill:** Funding acquisition (equal); methodology (equal); supervision (equal). **Deborah Arnott:** Data curation (equal); funding acquisition (equal). **Hazel Cheeseman:** Data curation (equal); funding acquisition (equal). **David Hammond:** Funding acquisition (equal); methodology (equal). **Jessica L. Reid:** Methodology (equal). **Pete Driezen:** Methodology (supporting). **Kimberly D'Mello:** Methodology (supporting). **Katherine East:** Conceptualization (equal); funding acquisition (equal); methodology (equal); supervision (equal).

## DECLARATION OF INTERESTS

D.H. has provided paid expert witness testimony on behalf of public health authorities in response to legal challenges from tobacco, vaping and cannabis companies, including standardized packaging laws for tobacco products. All authors declare no conflicts of interest.

## Supporting information


**Table S1.** Measure wording for outcome, vaping status, and smoking status in the ASH Youth Survey 2021.
**Table S2.** Social grade classification system, based on occupation of the chief income earner in the household.
**Figure S1.** Responses to e‐liquids pack images among youth aged 11–‐18, by vaping status and experimental condition; ASH‐Y 2021.
**Figure S2.** Responses to e‐liquids pack images among youth aged 11–18, by smoking status and experimental condition; ASH‐Y 2021.
**Table S3.** Multinomial logistic regression model of the associations between reporting no interest in trying or Do not Know and e‐liquid packaging condition, interaction by smoking status, ASH‐Y 2021 (*n* = 1628).
**Table S4.** Multinomial logistic regression model of the associations between reporting no interest in trying or Do not Know and e‐liquid packaging condition, interaction by vaping status, ASH‐Y 2021 (*n* = 1628).

## Data Availability

Data are available on reasonable request.
