# Supplementary material for: Association of fully branded, standardized packaging and limited flavor and brand descriptors of e‐liquids with interest in trying products among youths in Great Britain
Source: Addiction. 2025 Jan 13;120(4):620–8. doi: 10.1111/add.16763 (PMC11907326; doi:10.1111/add.16763)
Supplement: Supplementary file 1 — Table S1. Measure wording for outcome, vaping status, and smoking status in the ASH Youth Survey 2021. Table S2. Social grade classification system, based on occupation of the chief income earner in the household. Figure S1. Responses to e‐liquids pack images among youth aged 11–‐18, by vaping status and experimental condition; ASH‐Y 2021. Figure S2. Responses to e‐liquids pack images among youth aged 11–18, by smoking status and experimental condition; ASH‐Y 2021. Table S3. Multinomial logistic regression model of the associations between reporting no interest in trying or Do not Know and e‐liquid packaging condition, interaction by smoking status, ASH‐Y 2021 (n = 1628). Table S4. Multinomial logistic regression model of the associations between reporting no interest in trying or Do not Know and e‐liquid packaging condition, interaction by vaping status, ASH‐Y 2021 (n = 1628). [file ADD-120-620-s001.docx]

| **Table S1**. Measure wording for outcome, vaping status, and smoking status in the ASH Youth Survey 2021 |
| --- |
| INTEREST IN TRYING PRODUCTS SHOWN (OUTCOME) |
| ‘Which of the following products would *people your age* be most interested in trying?’  a Image of Slushie package  b Image of Puff Dragon package  c Image of Moreish Puff package  d ‘Prefer not to say’ (excluded)  e ‘None of these products’  f ‘Don’t know’  Coding  Interest in trying (a-c), vs no interest in trying (e), vs Don’t know (f) |
| VAPING STATUS |
| 1. ‘Have you ever heard of e-cigarettes? They are also sometimes called vapes, shisha pens or electronic cigarettes.’  a ‘Yes’  b ‘No’  c ‘Don’t know’  2: *Among those who had ever heard of e-cigarettes:* ‘Which ONE of the following is closest to describing your experience of e-cigarettes?’  a ‘I have never used an e-cigarette’  b ‘I have only tried an e-cigarette once or twice’  c ‘I use e-cigarettes sometimes, but no more than once a month’  d ‘I use e-cigarettes more than once a month, but less than once a week’  e ‘I use e-cigarettes more than once a week but not every day’  f ‘I use e-cigarettes every day’  g ‘I used e-cigarettes in the past but no longer do’  h ‘Don’t want to say’ (excluded)  Coding  Never (1b, 1c, 2a), Tried/former (2b, 2g), Current (2c-2f) |
| SMOKING STATUS |
| ’Which ONE of the following BEST applies to you?’  a ‘I have never smoked cigarettes, not even a puff or two’  b ‘I have only ever tried smoking cigarettes once’  c ‘I used to smoke sometimes but I never smoke cigarettes now’  d ‘I sometimes smoke cigarettes now but less than once a week’  e ‘I usually smoke between once and six cigarettes a week’  f ‘I usually smoke more than six cigarettes a week’  g ‘Don’t want to say’ (excluded)  Coding:  Never (a), Tried/former (b, c), Current (d-f) |

| **Table S2:** Social grade classification system, based on occupation of the chief income earner in the household | |
| --- | --- |
| A | Higher managerial, administrative and professional |
| B | Intermediate managerial, administrative and professional |
| C1 | Supervisory, clerical and junior managerial, administrative and professional |
| C2 | Skilled manual workers |
| D | Semi-skilled and unskilled manual workers |
| E | State pensioners, casual and lowest grade workers, unemployed with state benefits only |
| Social grade was based on the occupation of the chief income earner in the household, and was asked of the parents of those participants age 11–15, and directly of those participants age 16–18. | |

+ Group has small cell counts, data should be interpreted with caution.


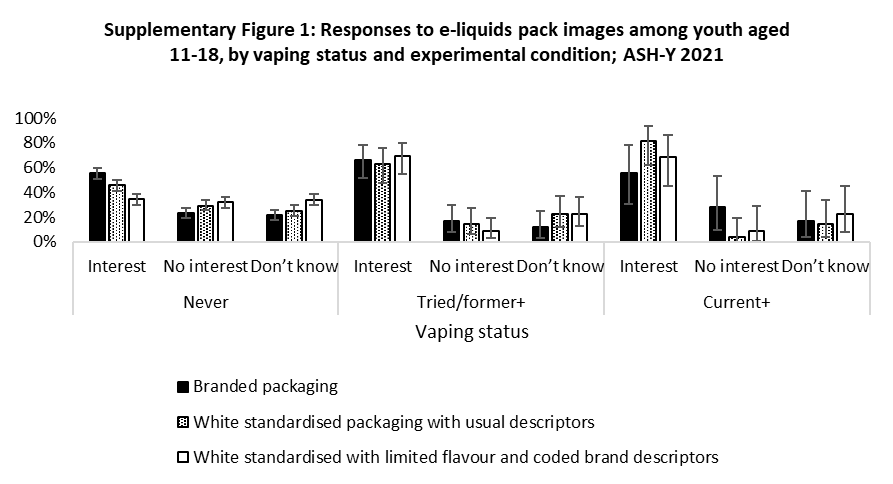


+ Group has small cell counts, data should be interpreted with caution.


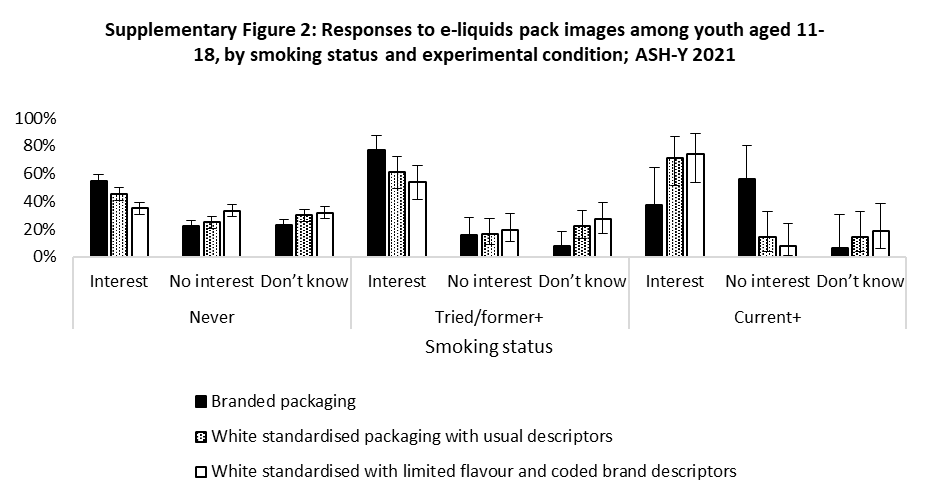


| **Table S3: Multinomial logistic regression model of the associations between reporting no interest in trying or Don’t Know and e-liquid packaging condition, interaction by smoking status, ASH-Y 2021 (n=1628)** | | | | | | | |
| --- | --- | --- | --- | --- | --- | --- | --- |
|  | Interest in trying any  e-liquid displayed (ref) | No interest in trying any e-liquid displayed | | | Don’t know | | |
|  | %(n) | %(n) | AOR(95%CI) | p | %(n) | AOR(95%CI) | p |
| **Ever smoked (N=262)** |  |  |  |  |  |  |  |
| Branded packaging (control) | 67.6(46) | 25.0(17) | 1 | Ref | 7.4(5) | 1 | Ref |
| White standardised packaging with usual descriptors | 64.0(64) | 16.0(16) | 0.74(0.32-1.71) | .255 | 20.0(20) | 2.91(1.01-8.43) | **.049** |
| White standardised with limited flavour and coded brand descriptors | 59.6(56) | 16.0(15) | 0.62(0.27-1.41) | .482 | 24.5(23) | 3.68(1.28-10.55) | **.015** |
|  |  |  |  |  |  |  |  |
| **Never smoked (N=1366)** |  |  |  |  |  |  |  |
| Branded packaging (control) | 54.7(257) | 22.3(105) | 1 | Ref | 23.0(108) | 1 | Ref |
| White standardised packaging with usual descriptors | 45.5(201) | 24.7(109) | 1.33(0.96-1.85) | .089 | 29.9(132) | 1.58(1.15-2.18) | **.005** |
| White standardised with limited flavour and coded brand descriptors | 35.0(159) | 33.3(151) | 2.40(1.74-3.32) | **<.001** | 31.7(144) | 2.24(1.62-3.09) | **<.001** |
|  |  |  |  |  |  |  |  |
| AOR= adjusted odds ratio  Analyses were adjusted for sex, age, socioeconomic status, vaping status (ever, never).  All data are unweighted | | | | | | | |

| **Table S4: Multinomial logistic regression model of the associations between reporting no interest in trying or Don’t Know and e-liquid packaging condition, interaction by vaping status, ASH-Y 2021 (n=1628)** | | | | | | | |
| --- | --- | --- | --- | --- | --- | --- | --- |
|  | Interest in trying any  e-liquid displayed (ref) | No interest in trying any e-liquid displayed | | | Don’t know | | |
|  | %(n) | %(n) | AOR(95%CI) | p | %(n) | AOR(95%CI) | p |
| **Ever Vaped (N=225)** |  |  |  |  |  |  |  |
| Branded packaging (control) | 63.4(45) | 19.7(14) | 1 | Ref | 16.9(12) | 1 | Ref |
| White standardised packaging with usual descriptors | 69.3(52) | 10.7(8) | 0.43(0.16-1.17) | .203 | 20.0(15) | 1.34(0.57-3.16) | .499 |
| White standardised with limited flavour and coded brand descriptors | 68.4(54) | 8.9(7) | 0.53(0.20-1.41) | .097 | 22.8(18) | 1.21(0.50-2.93) | .676 |
|  |  |  |  |  |  |  |  |
| **Never Vaped (N=1403)^a^** |  |  |  |  |  |  |  |
| Branded packaging (control) | 55.2(258) | 23.1(108) | 1 | Ref | 21.6(101) | 1 | Ref |
| White standardised packaging with usual descriptors | 45.6(213) | 25.1(117) | 1.34(0.97-1.84) | .080 | 29.3(137) | 2.50(1.81-3.46) | **<.001** |
| White standardised with limited flavour and coded brand descriptors | 34.3(161) | 33.9(159) | 2.48(1.80-3.21) | **<.001** | 31.8(1490 | 1.69(1.23-2.33) | **.001** |
|  |  |  |  |  |  |  |  |
| ^a^ Includes respondents who had never heard of e-cigarettes.  AOR= adjusted odds ratio  Analyses were adjusted for sex, age, socioeconomic status, smoking status (ever, never).  All data are unweighted | | | | | | | |
